# Biological Mechanism(s) Underpinning the Association between Antipsychotic Drugs and Weight Gain

**DOI:** 10.3390/jcm10184095

**Published:** 2021-09-10

**Authors:** Bruna Panizzutti, Chiara C. Bortolasci, Briana Spolding, Srisaiyini Kidnapillai, Timothy Connor, Mark F. Richardson, Trang T. T. Truong, Zoe S. J. Liu, Laura Gray, Jee Hyun Kim, Olivia M. Dean, Michael Berk, Ken Walder

**Affiliations:** 1 Institute for Innovation in Physical and Mental Health and Clinical Translation, IMPACT, School of Medicine, Deakin University, Geelong 3220, Australia; b.panizzuttiparry@deakin.edu.au (B.P.); chiara.b@deakin.edu.au (C.C.B.); briana.spolding@deakin.edu.au (B.S.); srisaiyini.kidnapillai@med.lu.se (S.K.); timothy.connor@deakin.edu.au (T.C.); truongtra@deakin.edu.au (T.T.T.T.); zoe.liu@deakin.edu.au (Z.S.J.L.); l.gray@deakin.edu.au (L.G.); jee.kim@deakin.edu.au (J.H.K.); o.dean@deakin.edu.au (O.M.D.); michael.berk@deakin.edu.au (M.B.); 2 Genomics Centre, School of Life and Environmental Sciences, Deakin University, Geelong 3220, Australia; m.richardson@deakin.edu.au; 3 Florey Institute for Neuroscience and Mental Health, University of Melbourne, Parkville 3052, Australia; 4 Department of Psychiatry, Royal Melbourne Hospital, University of Melbourne, Parkville 3052, Australia; 5 Centre of Youth Mental Health, University of Melbourne, Parkville 3052, Australia; 6 Orygen Youth Health Research Centre, Parkville 3052, Australia

**Keywords:** antipsychotics, weight gain, schizophrenia, lipid metabolism, bipolar disorder, metabolic syndrome, psychiatry, neuroscience, mental disorders

## Abstract

Weight gain and consequent metabolic alterations are common side-effects of many antipsychotic drugs. Interestingly, several studies have suggested that improvement in symptoms and adverse metabolic effects are correlated. We used next generation sequencing data from NT-2 (human neuronal) cells treated with aripiprazole, amisulpride, risperidone, quetiapine, clozapine, or vehicle control, and compared with the Pillinger P-score (ranked from 0 to 1, indicating greater increase in weight gain and related metabolic parameters) to identify the genes most associated with the drugs’ propensity to cause weight gain. The top 500 genes ranked for their correlation with the drugs’ propensity to cause weight gain were subjected to pathway analysis using DAVID (NIH). We further investigated transcription factors (TFs) that are more likely to regulate the genes involved in these processes using the prediction tool of key TFs from TRRUST. The results suggest an enrichment for genes involved in lipid biosynthesis and metabolism, which are of interest for mechanisms underpinning weight-gain. The list of genes involved in the lipid pathways that correlated with weight gain was enriched for genes transcriptionally regulated by *SREBF1* and *SREBF2*. Furthermore, quetiapine significantly increased the expression of *SREBF1* and *SREBF2* in NT-2 cells. Our results suggest that the effects of these antipsychotic drugs on lipid metabolism may be mediated, at least in part, via regulation of *SREBF1/SREBF2* expression, with evidence of a direct effect of quetiapine on the expression of *SREBF1/2*. The effects of antipsychotic drugs on lipid metabolism may influence white matter structure (therapeutic effect) and the risk of weight gain, lipid disturbances, and, consequently, metabolic syndrome (adverse effects). Understanding the different molecular effects of these drugs could inform a personalized medicine approach in treating patients with schizophrenia.

## 1. Introduction

Antipsychotic-Induced Weight Gain (AIWG) is a debilitating and common adverse effect of antipsychotic treatment, and negatively impacts on life expectancy, quality of life, treatment adherence, and the likelihood of developing the metabolic syndrome and type-2 diabetes [1]. Weight gain also adversely affects clinical outcomes such as readmission [1].

The molecular mechanisms behind AIWG remain mostly unknown. Several studies have investigated receptor binding profiles to explain the metabolic abnormalities related to weight-gain. Involvement of the dopamine-based reward stimulation might underlie the changes in food consumption [2]; preclinical models also indicate the involvement of histamine H1 receptors, through activation of the hypothalamic AMP-kinase signaling to increase food intake [3]. The participation of the serotonin 5-HT2 receptors in control of feeding behavior is well established [4], and more recently genetic polymorphisms in the serotonin receptor 2C were associated with an increase in AIWG [5]. With respect to receptor binding profiles olanzapine and clozapine, which block histamine H1, serotonin 5-HT2A/C and dopamine D2/3 receptors, cause the greatest weight gain [6]; while compounds with little or no histamine and serotonin affinity—lurasidone and aripiprazole for example—have a lower risk of weight gain [7]. The effects of neurohormones such as leptin, adiponectin, glucagon like protein 1 (GLP-1), and insulin suggests the involvement of the gut–brain axis might underly both the therapeutic and weight-gain sides [8,9]. 

Besides receptor binding profile and antipsychotic side-effects, the weight gain in patients treated with antipsychotics has been linked to individual and environmental characteristics. A meta-analysis identified 13 single-nucleotide polymorphisms that were significantly associated with AIWG [10], and the pharmacogenomics associated with drug-induced weight gain was reviewed by Sneha Singh and colleagues [11] and Soria-Chacartegui [12]. Children and adolescents seem to be especially vulnerable to AIWG [13,14]. Increased risk for AIWG was also linked to first exposure to antipsychotic medication, longer use of antipsychotics, baseline weight, gender, and other individual characteristics [6,15,16].

In an effort to understand the effects of different antipsychotics on the metabolic alterations that occur in patients treated with these drugs, Pillinger and colleagues performed a systematic review and network meta-analysis comparing 18 antipsychotic medications [17]. One of the parameters investigated was change in body weight. In 83 studies included in the analysis, with 18,750 patients using antipsychotic medication for a median of 6 weeks, and 4210 patients in the placebo group, the authors were able to rank the antipsychotics for the degree of weight-gain using a P-score with a scale of 0 to 1, where the higher P-score indicated greater increase in weight-gain.

Therefore, using the P-score generated by the Pillinger [17] network meta-analysis and differential gene expression in neuronal like cells treated with antipsychotics we aimed to identify pathways or gene sets linked to biological mechanism(s) underpinning the propensity of antipsychotic drugs to cause weight gain.

## 2. Materials and Methods

### 2.1. Cell Culture

NT2 human teratocarcinoma cells (CVCL_0034, ATCC, Manassas, VA, USA) were cultured as previously described [18]. Briefly, the cells were maintained in standard cell culture media and then differentiated into neuronal-like cells using 1 × 10^−5^ M retinoic acid (Sigma-Aldrich, Sydney, Australia) for 28 days with media refreshed every 2–3 days. For experiments, cells were seeded at 2 × 10^5^ cells/well (24-well plates) and treated with mitotic inhibitors (1 µM cytosine and 10 µM uridine; Sigma-Aldrich) every 2–3 days for a total of seven days. The cells were then treated with aripiprazole (0.1 µM), amisulpride (10 µM), clozapine (10 µM), quetiapine (50 µM), or risperidone (0.10 µM) for 24 h. Treatment doses and intervals were determined in previous dose–response studies in our lab, so that, when used in combination, no single drug dominated the effect on gene expression or affected cell viability; and were carried out throughout the following projects [18,19]. All drugs were purchased from Sigma-Aldrich (Sydney, Australia). Vehicle control cells were treated with 0.2% dimethyl sulfoxide (DMSO).

### 2.2. Gene Expression

Following the 24-h drug treatment, cells were harvested using Trizol, and total RNA was extracted using RNeasy^®^ mini kits (Qiagen, Melbourne, Australia) and quantified by spectrophotometry (NanoDrop 1000 Thermo Fisher Scientific, Waltham, MA, USA). The quality of the extracted RNA was evaluated using an Agilent 2100 Bioanalyzer (Agilent Technologies, Melbourne, Australia). RNAseq libraries were prepared from 1ug of total RNA using a TruSeq RNA samples Preparation kit (Illumina, Victoria, Australia). The libraries were sequenced using a HiSeq 2500 flow cell (50 bp single end reads; Illumina) according to the manufacturer’s instructions.

### 2.3. Genome-Wide Gene Expression Analysis

The raw data were obtained in fastq format and processed using the Deakin Genomics Centre RNAseq alignment and expression quantification pipeline (https://github.com/m-richardson/RNASeq_pipe, accessed on 1 July 2017). In summary, this involves: Raw read quality filtering and adapter trimming (ILLUMINACLIP:2:30:10:4, SLIDINGWINDOW:5:20, AVGQUAL:20 MINLEN:36) with Trimmomatic v35 [20], and alignment to the reference genome using STAR v2.5 in 2-pass mode (Human genome version GRCh38) [21]. The expression was quantified at the gene level, and individual sample counts were collated into a m × n matrix for differential abundance testing. Normalization (TMM) and removal of low expressed gene were performed using edgeR [22] in R [23] following the edgeR manual (<1 cpm in n samples, where n is the number of samples in the smallest group for comparison). Differential gene expression analysis was assessed using edgeR in R, and the Benjamini–Hochberg [24] corrected *p*-values were calculated to account for multiple testing. Genes with corrected *p*-values of <0.05 were considered to be differentially expressed (Supplementary Table S1).

### 2.4. Pathway Analysis

We used parametric correlation to identify the genes most associated with the drug’s propensity to cause weight-gain. Within the list of differentially expressed genes (logFC) and the P-scores calculated by Pillinger (Table 1) we identified the top 500 genes that were positively correlated with the propensity to cause weight gain and subjected these genes to pathway analysis using the Database for Annotation, Visualization and Integrated Discovery (DAVID; National Institutes of Health) [25].

### 2.5. Transcriptional Regulatory Relationships Unravelled by Sentence-Based Text-Mining (TRRUST)

TRRUST is a manually curated database for transcriptional regulatory networks that can predict key transcription factors (TF) regulating gene expression [26]. The differentially expressed genes in the pathways of interest were submitted for analysis using TRRUST.

## 3. Results

### 3.1. DAVID Pathways

The top enriched pathways resulting from the top 500 genes correlated with the propensity of the antipsychotics to cause weight gain are listed in Table 2. Although the Benjamini adjusted *p*-value was not significant (<0.05), there appears to be enrichment for genes involved in lipid and cholesterol metabolism, which are of interest for mechanisms underpinning weight-gain.

### 3.2. TRRUST

Next, we submitted the differentially expressed genes in the pathways of interest, i.e., lipid biosynthesis and lipid metabolism (Table 3) to TRRUST.

The list of lipid metabolism genes that were correlated with weight gain was enriched for genes that are transcriptionally regulated by *SREBF1* (FDR = 1.76 × 10^−5^) (Table 4). This suggests that the antipsychotic drugs’ effects on lipid metabolism may be mediated, at least in part, via regulation of *SREBF1/SREBF2* expression.

### 3.3. Antipsychotic Drug Effects on SREBFs

We also investigated whether the antipsychotic drugs used to treat the NT-2 cells had effects on the gene expression of SREBFs (Table 5). Quetiapine was the only drug to significantly increase the expression of *SREBF1* and *SREBF2* (FDR = 1.03 × 10^−11^ and FDR = 10.9 × 10^−22^). 

## 4. Discussion

We demonstrated that five commonly prescribed antipsychotic drugs change the expression of genes involved in the lipid biosynthesis and metabolic pathways, and that these genes might be regulated by *SREBF1* and *SREBF2*. SREB proteins are transcription factors that play a key role in cholesterol biosynthesis influencing both uptake and fatty acid biosynthesis as well as upregulating the synthesis of sterol biosynthesis enzymes [27].

Our results are in line with previous experiments demonstrating the upregulation of genes regulated by SREB transcription factors after antipsychotic drug treatment in glial cells [28] and central nervous system related cell lines [29]. In glial cells, *HMGCR*, *FASN*, *SREBP-1*, and *SREBP-2* expression was increased after treatment with haloperidol and clozapine [28]. Subsequent studies showed effects of chlorpromazine, haloperidol, olanzapine, risperidone, and ziprasidone on *SREBPs* and downstream genes, in GaMg and CCF-STTG1 glial cells, HCN2 cortical neurons and SH-SY5Y neuroblastoma cell lines [29]. In the context of the CNS these findings can be interpreted in line with the abnormalities in myelination and white matter identified in patients with schizophrenia [30], suggesting that antipsychotics might target symptoms at least in part through lipogenic activation and consequently increase myelination [31,32]. 

These results were also confirmed in adipocytes. Studies showed that olanzapine induced adipogenesis through the overexpression of genes regulated by *SREBF1* [33]; and clozapine, olanzapine, and risperidone increased lipogenesis through Insig/SCAP/SREBP signalling [34]. This suggests that the therapeutic effect of antipsychotics on the CNS are associated with systemic effects that are at least in part responsible for the side-effects related to weight-gain and metabolic syndrome [35]. 

The association of polymorphisms in the *SREBF1* and *SREBF2* genes with schizophrenia was first reported in 2010 and has been replicated in three independent samples [36]. Further corroborations were reported in a Chinese cohort treated with clozapine; with two *SREBF2* SNPs being associated with increased risk for the drug-induced metabolic syndrome [37]. The same group later reported an association of SREF1 and SCAP (SREBP cleavage-activation protein) SNPs with elevated risk of drug-induced metabolic syndrome in people with schizophrenia [38]. Altogether these studies point towards interindividual variances for AIWG that may be associated with genetic variation in *SREBF* genes.

Our results add to the existing knowledge by showing that aripiprazole, amisulpride, and quetiapine also affect the expression of genes involved in lipid and cholesterol pathways, with quetiapine having a direct effect on the expression of *SREBF1* and *SREBF2*. 

Baptista and colleagues investigated the efficacy of weight loss interventions [39], with the meta-analysis showing that amantadine, metformin, reboxetine, sibutramine and topiramate were partially effective in reducing AIWG. This was corroborated recently in a study showing that treatment augmentation with metformin and GLP-1RA cause reduction in weight with minimal reports of severe side effects [40]. Later analysis suggested that metformin had a greater effect in weight loss [41]. Due to the differences between antipsychotics in terms of the propensity to cause weight gain, a recent study investigated whether switching to an antipsychotic with lesser metabolic effects, such as aripiprazole, amisulpride, and ziprasidone [15] would be beneficial for weight loss. The results indicated that weight gain was mild in patients that did change treatments, although antipsychotic switch did not necessarily result in weight loss. 

We acknowledge some limitations of our study. Pillinger et al. [17] used a comprehensive list of drugs in their study, however due to resource limitations, we prioritised the five drugs used in this study because they are commonly prescribed and thought to be mechanistically diverse. We tested a single dose of each drug and measured acute effects; therefore, these finds do not capture effects of chronic administration or drug–drug interactions. In addition, our experiments used neuronal-like cells without inducing of any disease model, underlying pathophysiological processes specific for each disease might influence the drug effects.

Here we showed that the propensity of antipsychotic drugs to cause weight gain was associated with differential effects on lipid metabolic genes at a transcriptional level independent of receptor binding profile, and that SREBF transcription factors may play a role in these effects. Taken together, the data on efficacy [7] adverse events and risk factors [17], and knowledge of the underlying pathways can inform a personalised medicine approach for treating patients with schizophrenia.

## Figures and Tables

**Table 1:** Antipsychotic Propensity to Cause Weight-Gain P-Scores.

Drug	P-Score
Aripiprazole	0.26
Amisulpride	0.41
Risperidone	0.58
Quetiapine	0.65
Clozapine	0.90

P-score: value resulted from network metanalysis performed by Pillinger and colleagues [17]. P-score ranges from 0 to 1, with higher scores indicating a greater degree of weight gain and metabolic disturbance.

**Table 2:** Top Pathways Enriched in Genes Positively Correlated with the Drugs Propensity to Cause Weight Gain.

Category	Term	Count	*p*-Value	FDR
UP_KEYWORDS	Lipid biosynthesis	13	0.00020	0.054
UP_KEYWORDS	Lipid metabolism	23	0.00031	0.054
UP_KEYWORDS	Cholesterol biosynthesis	5	0.0007	0.084
UP_KEYWORDS	Steroid biosynthesis	6	0.0010	0.088
GOTERM_CC_DIRECT	Endoplasmic reticulum	34	0.0012	0.38
UP_KEYWORDS	Cholesterol metabolism	7	0.0016	0.10
UP_KEYWORDS	Alternative splicing	268	0.0017	0.10
GOTERM_BP_DIRECT	Cholesterol biosynthesis process	6	0.0019	0.41
KEGG_PATHWAY	Lysosome	10	0.0019	0.41
UP_KEYWORDS	Sterol biosynthesis	5	0.0021	0.11

Count = number of genes in the pathway; DR = false discovery rate.

**Table 3:** Correlations between Differentially Expressed Genes in the Lipid Biosynthesis and Lipid Metabolism Pathways with P-Score.

Gene	r^2^	*p*-Value
LIPID BIOSYNTHESIS		
PCYT2	0.93	0.0032
ACACA	0.69	0.061
HMGCR	0.66	0.074
MCAT	0.66	0.074
FDPS	0.63	0.087
DHCR24	0.62	0.093
LIPID METABOLISM		
SREBF1	0.96	0.00091
PCYT2	0.93	0.0032
PCSK9	0.76	0.038
LPIN1	0.73	0.045
ACACA	0.69	0.061
PLIN1	0.67	0.068
MCAT	0.66	0.074
HMGCR	0.66	0.074
FDPS	0.63	0.087
DHCR24	0.62	0.093

r^2^ = parametric correlation between different expressed genes and P-score.

**Table 4:** TRRUST Transcription Factors Regulating Lipid Biosynthesis and Metabolism Genes.

Key TF	Description	*p*-Value	FDR	List of Overlapped Genes
LIPID BIOSYNTHESIS				
*SREBF2*	sterol regulatory element binding transcription factor 2	5.84 × 10^−5^	5.84 × 10^−5^	HMGCR, FDPS
LIPID METABOLISM				
*SREBF2*	sterol regulatory element binding transcription factor 2	2.31 × 10^−6^	1.38 × 10^−5^	HMGCR, PCSK9, FDPS
SREBF1	sterol regulatory element binding transcription factor 1	5.88 × 10^−6^	1.76 × 10^−5^	LPIN1, PCSK9, FDPS
NFYC	nuclear transcription factor Y, gamma	0.00013	0.000261	FDPS, LPIN1
RELA	v-rel reticuloendotheliosis viral oncogene homolog A (avian)	0.0598	0.0726	PCYT2, PLIN1
NFKB1	nuclear factor of kappa light polypeptide gene enhancer in B-cells 1	0.0605	0.0726	PCYT2, PLIN1
SP1	Sp1 transcription factor	0.129	0.129	SREBF1, DHCR24

TF = transcriptional factor; FDR = false discovery rate.

**Table 5:** Antipsychotic Effect of SREB Gene Expression.

	*SREBF1*				*SREBF2*			
	logFC	logCPM	*p*-Value	FDR	logFC	logCPM	*p*-Value	FDR
Quetiapine	0.53	5.35	3.11 × 10^−13^	1.03 × 10^−11^	0.26	8.81	1.01 × 10^−24^	1.09 × 10^−22^
Amisulpride	−0.15	4.93	0.17	0.50	−0.10	8.50	0.0499	0.30
Aripiprazole	−0.23	4.93	0.10	0.34	−0.16	8.49	0.025	0.18
Clozapine	0.23	5.23	0.026	0.20	0.05	8.63	0.41	0.72
Risperidone	0.05	7.97	0.22	0.83	−0.06	8.53	0.17	0.80

Log FC = logarithmic fold change; logCPM = logarithmic count per million; FDR = false discovery rate.

## Data Availability

Data available from author upon request.

## Supplementary Materials

The following are available online at https://www.mdpi.com/article/10.3390/jcm10184095/s1, Table S1: Differentially expressed genes per treatment.
